# Endothelium Activation during Severe Yellow Fever Triggers an Intense Cytokine-Mediated Inflammatory Response in the Liver Parenchyma

**DOI:** 10.3390/pathogens11010101

**Published:** 2022-01-15

**Authors:** Fábio Alves Olímpio, Luiz Fábio Magno Falcão, Marcos Luiz Gaia Carvalho, Jeferson da Costa Lopes, Caio Cesar Henriques Mendes, Arnaldo Jorge Martins Filho, Carlos Augusto Moreira da Silva, Vanessa do Socorro Cabral Miranda, Lais Carneiro dos Santos, Fellipe Souza da Silva Vilacoert, Ana Cecília Ribeiro Cruz, Vanessa Costa Alves Galúcio, Raimunda do Socorro da Silva Azevedo, Lívia Caricio Martins, Maria Irma Seixas Duarte, Jorge Rodrigues de Sousa, Pedro Fernando da Costa Vasconcelos, Juarez Antônio Simões Quaresma

**Affiliations:** 1 Núcleo de Medicina Tropical, Universidade Federal do Pará, Belém 66050-540, Brazil; f.olimpiomilitar@gmail.com; 2 Seção de Arbovirologia e Febres Hemorrágicas, Instituto Evandro Chagas, Ministério da Saúde, Ananindeua 67030-000, Brazil; fabiofalcao@uepa.br (L.F.M.F.); marcosgaia@outlook.com (M.L.G.C.); jefersonchaz@hotmail.com (J.d.C.L.); arnaldofilho@iec.gov.br (A.J.M.F.); vanessacabralmiranda@gmail.com (V.d.S.C.M.); laiscarneiros@gmail.com (L.C.d.S.); fellipe.villa.07@gmail.com (F.S.d.S.V.); anacecilia@iec.gov.br (A.C.R.C.); raimundaazevedo@iec.gov.br (R.d.S.d.S.A.); liviamartins@iec.gov.br (L.C.M.); krekrodrigues@gmail.com (J.R.d.S.); 3 Centro de Ciências Biológicas e da Saúde, Universidade do Estado do Pará, Belém 66087-670, Brazil; caio_henriques12@hotmail.com; 4 Departamento de Patologia, Faculdade de Medicina, Universidade de São Paulo, São Paulo 05508-070, Brazil; miduarte@usp.br; 5 Coordenação de Vigilância em Saúde, Secretaria de Saúde Pública do Estado do Pará, Belém 66093-677, Brazil; cmoreira@gmail.com; 6 Curso de Biomedicina, Faculdade Cosmopolita, Belém 66615-005, Brazil; vanessagalucio@gmail.com

**Keywords:** yellow fever, liver, vascular endothelium, adhesion molecule

## Abstract

Yellow fever (YF) is a pansystemic disease caused by the yellow fever virus (YFV), the prototype species of the family *Flaviviridae* and genus *Flavivirus*, and has a highly complex host-pathogen relationship, in which endothelial dysfunction reflects viral disease tropism. In this study, the in situ endothelial response was evaluated. Liver tissue samples were collected from 21 YFV-positive patients who died due to the disease and five flavivirus-negative controls who died of other causes and whose hepatic parenchyma architecture was preserved. Immunohistochemical analysis of tissues in the hepatic parenchyma of YF cases showed significantly higher expression of E-selectin, P-selectin, intercellular adhesion molecule-1, vascular cell adhesion molecule-1, and very late antigen-4 in YFV-positive cases than in flavivirus-negative controls. These results indicate that endothelium activation aggravates the inflammatory response by inducing the expression of adhesion molecules that contribute to the rolling, recruitment, migration, and construction of the inflammatory process in the hepatic parenchyma in fatal YF cases.

## 1. Introduction

Yellow fever (YF) is a mosquito-borne disease caused by the yellow fever virus (YFV), an arbovirus belonging to the family *Flaviviridae* and genus *Flavivirus*. In recent years, the disease has drawn attention due to the risk of re-emergence of urban YF [1,2,3]. Between 2016–2019, Brazil experienced a major epidemic in the country [3]. YF is considered the original viral haemorrhagic fever that induces a pansystemic response in severe clinical presentations [3,4]. The pathophysiological processes determining the clinical course of the disease depend on the viral properties and immune response pattern of the host, which can determine the appearance of severe lesions in various organs and tissues, such as the liver, heart, kidneys, and vascular endothelium. In the liver, histopathological changes are induced by the direct viral cytopathic effect through changes in the sinusoidal microvasculature and tissue immune response [5,6,7].

Considering that one of the immune evasion strategies triggered by YFV is the modification of the behaviour of the vascular endothelium in the liver as its central target, it is unexpected that only a few studies have demonstrated how the expression of adhesion molecules can contribute to the rolling, recruitment, migration, and construction of the inflammatory process in the organ. Thus, we investigated the role of selectins (E and P), intercellular adhesion molecule-1 (ICAM-1), vascular cell adhesion molecule-1 (VCAM-1), and very late antigen-4 (VLA-4) in situ in the liver parenchyma of severe fatal cases of human YF.

## 2. Methods

### 2.1. Patients, Samples, and Diagnostic Confirmation

Liver tissue samples were collected from 26 patients. Among them, 21 fatal cases of human YF had positive results for a quantitative reverse transcription-polymerase chain reaction and/or an immunohistochemical assay, and five patients tested negative for YFV and other flaviviruses circulating in Brazil and showed a preserved hepatic architecture. The total RNA was extracted from the tissue using the ReliaPrepTM FF-PE total RNA Miniprep System (Promega) according to the manufacturer’s recommendations. Real-time assays were performed according to the adapted protocol of Ferreira et al. [5]. For histopathological analysis, 5 µM sections were cut from paraffin-embedded tissue samples and stained with haematoxylin and eosin.

### 2.2. Ethical Aspects

The methods used in this study were in accordance with the relevant guidelines and regulations of the Ministry of Health Ethics Committee. Moreover, the experimental protocols used in the present study were approved by the Research Ethics Committee of the Evandro Chagas Institute (IEC) (decision number 2462.701), Ananindeua, Pará, Brazil, and complied with the recommendations of the National Health Council No. 466/2012.

### 2.3. Immunohistochemical Assay

The labelled streptavidin biotin (LSAB) detection method was used for immunostaining of tissues with antibodies against E-selectin (Novus Biologicals, Centennial, CO, USA, NBP1-40109), P-selectin (Novus Biologicals, NBP2-22046), ICAM-1 (RD Systems, Minneapolis, MN, USA, BBA19), VCAM-1 (Abcam, Cambridge, UK, ab 134047), and VLA-4 (Novus Biologicals, NB110-55525). For immunostaining, paraffin-embedded tissue samples were dewaxed in xylol and hydrated in ethyl alcohol at concentrations of 90%, 80%, and 70% in this sequence. Endogenous peroxidase was blocked using 3% hydrogen peroxide for 45 min. Antigenic recovery was performed using citrate buffer (pH 6.0) for 20 min at 90 °C, and non-specific proteins were blocked using10% skimmed milk, which was concentrated for 30 min. The primary antibodies were diluted in 1% bovine serum albumin for 14 h, and then the biotinylated secondary antibody LSAB (DakoCytomation, Glostrup, Denmark) was added for 30 min at 37 °C, followed by streptavidin peroxidase (DakoCytomation) for 30 min at 37 °C. The reaction was developed using a chromogenic solution composed of 0.03% diaminobenzidine and 3% hydrogen peroxide, and the tissue sections were counterstained with Harris’ haematoxylin for 1 min. Finally, the sections were dehydrated in an increasing ethanol series and cleared in xylene.

### 2.4. Quantitative Analysis and Photodocumentation

The slides prepared from the samples were analysed using an Axio Imager Z1 microscope (Zeiss, Oberkochen, Germany). Immunostaining was evaluated quantitatively by randomly selecting 40 fields at high magnification (400×), namely ten in the centrolobular zone (Z3), ten in the midzonal zone (Z2), ten in the periportal zone (Z1), and ten in the portal tract (PT), in both positive and negative cases. Each field was subdivided into 10 × 10 subdivision cross-sections delimited by a grid, comprising an area of 0.0625 mm^2^.

### 2.5. Statistical Analysis

The data obtained were stored in an electronic spreadsheet and analysed using GraphPad Prismsoftware (GraphPad Software Inc., San Diego, CA, USA). The numerical variables were expressed as the mean, median, standard deviation, and variance. One-way analysis of variance and Tukey tests were applied, and significance was set at *p* ≤ 0.05.

## 3. Results

**Figure 1:**
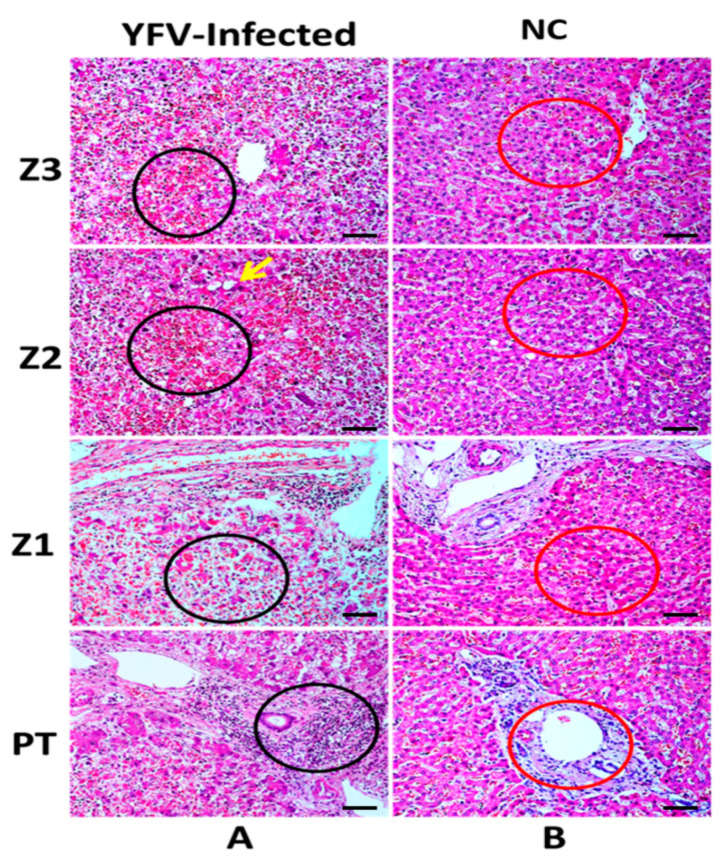
Histopathological analysis of the acini zones in the liver of fatal yellow fever human cases and normal controls (flavivirus negative using quantitative reverse transcription-polymerase chain reaction and/or immunohistochemistry). Z3, Z2, Z1, and PT in the hepatic parenchyma of fatal cases affected by YFV and control. (**A**) Area of necrosis with haemorrhagic foci in A-Z3, A-Z2, and A-Z1 (black circles). Steatosis (yellow arrow) in Z2. Massive presence of inflammatory infiltrates in the PT (black circle). (**B**) Preservation of the hepatic parenchyma (Z3, Z2, Z1, and PT) in control cases (red circles). Z3: Centrolobular zone; Z2: Midzonal zone; Z1: Periportal zone; PT: Portal tract; *p* ≤ 0.0001. 400×, scale bar (20 µm).

Steatosis (macro-and microvesicular) was one of the main hepatic changes observed and was present in the three lobular areas, predominantly in zone 2 (Z2). Councilman bodies, as well as lytic and coagulative necrosis, were recurrent in the three zones, most frequently in Z2. In addition, the inflammatory infiltrate was found in large quantities in the portal tract (PT) and in a smaller amount in the acini, with disproportionate intensity to the degree of impairment of the liver parenchyma, being more intense in Z2. Additionally, changes in the sinusoidal endothelium were observed, especially in Z2, when compared to the hepatic acinus zones (Figure 1A).

### Analysis of Endothelial Immunomarkers

**Figure 2:**
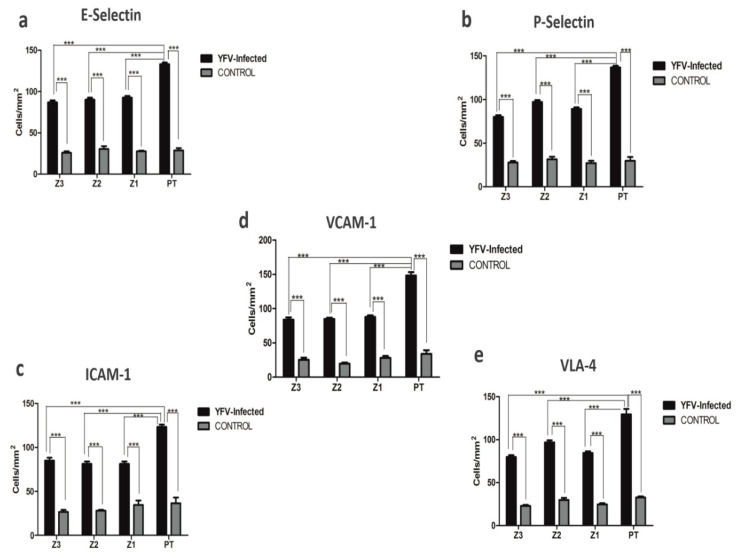
Graphics for immunohistochemical analysis positive for (**a**) E-selectin, (**b**) P-selectin, (**c**) intercellular adhesion molecule-1 (ICAM-1), (**d**) vascular cell adhesion molecule-1 (VCAM-1), and (**e**) very late antigen-4 (VLA-4) in zones Z3, Z2, Z1, and PT in the hepatic parenchyma of yellow fever fatal cases and in yellow fever negative controls. Z3: Centrolobular zone; Z2: Midzonal zone; Z1: Periportal zone; PT: Portal tract; *** *p* ≤ 0.0001.

**Figure 3:**
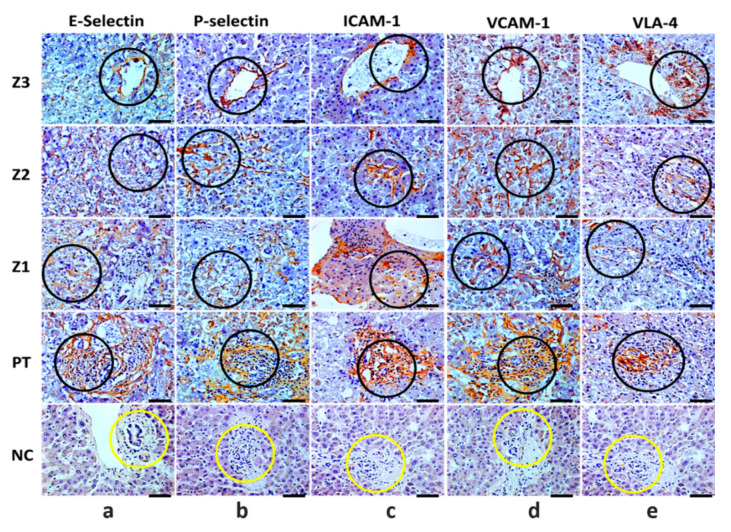
Immunohistochemical analysis positive for E-selectin, P-selectin, intercellular adhesion molecule-1 (ICAM-1), vascular cell adhesion molecule-1 (VCAM-1), and very late antigen-4 (VLA-4) in zones Z3, Z2, Z1, and PT in the hepatic parenchyma of yellow fever fatal cases and in yellow fever negative controls. Immunostaining for E-selectin (**a**), P-selectin (**b**), ICAM-1 (**c**), VCAM-1 (**d**), and VLA-4 (**e**) in the endothelial cells (black circle) of -Z3, -Z2, -Z1 and inflammatory infiltrate (PT) (black circle). Z3: Centrolobular zone; Z2: Midzonal zone; Z1: Periportal zone; PT: Portal tract; *p* ≤ 0.0001. In negative controls (NC): Light labelling for E-selectin and preservation of the PT (yellow circle), and absence of labelling and preservation of the PT for P-selectin, ICAM-1, VCAM-1, and VLA-4 (yellow circles). 400×, scale bar (20 µm).

The major endothelial markers E-selectin, P-selectin, ICAM-1, VCAM-1, and VLA-4 were expressed in all human cases of fatal YF, and their quantitative analysis showed significant differences when compared to the control samples. Furthermore, it was observed that in the hepatic acinus, Z2 predominated in the expression of adhesion molecules that accompany PT (Table 1) (Figure 2a–e). It is worth noting that in the hepatic acinus, immunomarking of endothelial markers was recurrent mainly in endothelial cells (flattened appearance) and was massive in the PT in the presence of inflammatory infiltrates in positive cases compared to the control samples (Figure 3a–e).

## 4. Discussion

The immunological mechanisms related to the evolution of YF in humans are not yet fully understood [6]. It is known that vascular permeability in severe YF is caused by the interaction of several factors, including the action of inflammatory cytokines and the expression of endothelial markers [6]. The investigation of histopathological changes in the fatal cases of human YF analysed in our study reinforces the fact that in the liver parenchyma, tissue damage is accentuated, where the mechanisms of cell damage are consistent with the phenomenon of cell death associated with necrosis and apoptosis progression and are predominant in the midzonal zone (Z2). In this context, the response induced by cytokines, such as tumour necrosis factor-alpha (TNF-α), interferon-gamma, transforming growth factor-beta (TGF-β), Fas ligand, as well as CD4 T and CD8 T lymphocytes, natural killer cells, macrophages, and neutrophils are fundamental for triggering this process [8,9]. In addition, a striking feature of the fatal cases is the presence of an inflammatory infiltrate, where the vascular endothelium contributes significantly to the recruitment of defence cells in the hepatic acinus, mainly in the PT. This was evidenced by the analysis of the expression of endothelial markers involved in the study.

In the case of selectins (E and P), ICAM-1, VCAM-1, and VLA-4, it is important to highlight that in previous studies with other major flaviviruses associated with haemorrhagic diseases, such as dengue viruses, the expression of the markers correlates positively with the activity of interleukin 1-alpha, TNF-α, and nuclear factor kappa-light-chain-enhancer of activated B cells [10,11]. The activation of the vascular endothelium mechanism causes leukocyte recruitment to sites of inflammation to establish interactions among leukocytes, platelets, and endothelial cells.

In this scenario, to inhibit the viral replication process, a varied set of defence cells generates a strong response characterised as a cytokine storm that causes tissue damage and lytic or coagulative necrosis. Interestingly, the presence of leukocytes in the damaged environment aggravates tissue destruction, considering that the extravasation of the enzymatic content released by necrotic cells potentiates the inflammatory response. In the immunopathogenesis of haemorrhagic YF, the type 1 T-helper profile is the main regulator of the pro-inflammatory response in VCAM-1, which is a major regulator of leukocyte adhesion and transendothelial migration through interaction with alpha-4 beta-1 integrin (VLA-4) [9,11,12].

In severe haemorrhagic diathesis, as observed in YF, an extremely harmful environment is built with the marked presence of an altered (lesion in the) vascular endothelium, which plays a role in adhesion molecules, cytokine storm, and the intense recruitment of defence cells triggered in the organ in response to viral aggression. Taken together, with the intense and severe in situ damage, our results strongly suggest the occurrence of a massive hepatic propagation injury that culminates in consumptive coagulopathy and haemorrhagic manifestations classic to the fatal outcome of the disease [13].

## Authors Contribution

L.F.M.F, J.R.d.S, J.A.S.Q. and P.F.d.C.V. designed the study; M.L.G.C., J.R.d.S., J.d.C.L., C.C.H.M., A.J.M.F., F.A.O., C.A.M.d.S., V.d.S.C.M., L.C.d.S., F.S.d.S.V., R.d.S.d.S.A., A.C.R.C., V.C.A.G., M.I.S.D. and L.C.M. performed lab tests; M.I.S.D., J.A.S.Q. and P.F.C.V. furnished reagents; L.F.M.F., J.R.d.S., M.I.S.D., J.A.S.Q. and P.F.d.C.V. drafted the manuscript. All authors have read and agreed to the published version of the manuscript.

## Figures and Tables

**Table 1:** Quantitative analysis of endothelial markers in the hepatic parenchyma (Z3, Z2, Z1, and PT) in fatal cases of humans affected by yellow fever virus (YFV) compared to control.

Markers (Cells/mm^2^)	Z3	Tukey (*p* ≤ 0.05)	Z2	Tukey (*p* ≤ 0.05)	Z1	Tukey (*p* ≤ 0.05)	PT	Tukey (*p* ≤ 0.05)	ANOVA (*p* ≤ 0.05)
E-Selectin Control	86.93 ± 8.791 27.84 ± 3.683	***	90.13 ± 17.91 30.40 ± 7.756	***	92.65 ± 9.314 27.52 ± 2.086	***	133.0 ± 9.828 28.80 ± 6.197	***	***
P-Selectin Control	80.15 ± 11.15 28.16 ± 3.505	***	97.07 ± 10.07 31.68 ± 6.238	***	89.22 ± 8.110 27.20 ± 5.657	***	136.8 ± 8.960 29.76 ± 9.772	***	***
ICAM-1 Control	84.95 ± 15.43 26.56 ± 5.378	***	81.30 ± 12.54 28.16 ± 2.147	***	81.30 ± 12.54 34.56 ± 11.40	***	123.3 ± 12.64 36.48 ± 14.59	***	***
VCAM-1 Control	83.98 ± 15.24 25.28 ± 7.102	***	84.95 ± 8.094 19.52 ± 4.141	***	87.92 ± 13.36 28.16 ± 6.155	***	148.5 ± 21.50 33.92 ± 11.78	***	***
VLA-4 Control	79.77 ± 9.749 24.96 ± 5.258	***	96.84 ± 11.09 29.76 ± 5.611	***	84.42 ± 9.437 24.64 ± 3.318	***	129.5 ± 28.83 32.64 ± 3.119	***	***

Z3: Centrolobular zone; Z2: Midzonal zone; Z1: Periportal zone; PT: Portal tract; *** *p* ≤ 0.0001.
